# The promise of GaAs 200 in small-angle neutron scattering for higher resolution

**DOI:** 10.1107/S1600576724007246

**Published:** 2024-08-27

**Authors:** A. Magerl, H. Lemmel, M. Appel, M. Weisser, U. Kretzer, M. Zobel

**Affiliations:** a Biophysics Group, Physics Department Friedrich Alexander Universität Erlangen-Nürnberg Henkestrasse 91 91052Erlangen Germany; b JCNS-3: Neutron Analytics for Energy Research Forschungszentrum Jülich GmbH Wilhelm-Johnen-Strasse 52428Jülich Germany; c Atominstitut TU Wien Stadionallee 2 1020Wien Austria; d Institut Laue–Langevin 71 Avenue des Martyrs 38000Grenoble France; e Crystallography and Structural Physics, Physics Department Friedrich-Alexander-Universität Erlangen-Nürnberg Staudtstrasse 3 91058Erlangen Germany; f Freiberger Compound Materials GmbH, Am Junger-Löwe-Schacht 5,09599Freiberg, Germany; g Institute of Crystallography RWTH Aachen University Jägerstrasse 17-19 52066Aachen Germany; Uppsala University, Sweden; The European Extreme Light Infrastucture, Czechia

**Keywords:** ultra-small-angle neutron diffraction, USANS, Bonse–Hart diffractometer, GaAs 200

## Abstract

The *Q* resolution in Bonse–Hart double-crystal diffractometers is determined for a given Bragg angle by the value of the crystallographic structure factor. To date, the reflections Si 220 or Si 111 have been used exclusively in neutron scattering, which provide resolutions for triple-bounce crystals of about 2 × 10^−5^ Å^−1^ (FWHM). The Darwin width of the GaAs 200 reflection is about a factor of 10 smaller, offering the possibility of a *Q* resolution of 2 × 10^−6^ Å^−1^ provided crystals of sufficient quality are available. This article reports a feasibility study with single-bounce GaAs 200, yielding a *Q* resolution of 4.6 × 10^−6^ Å^−1^, six times superior in comparison with a Si 220 setup.

## Abstract 2

A Bonse–Hart camera using a Bragg reflection with a small crystallographic structure factor will enable one to access correspondingly low *Q* values in small-angle diffraction. This is demonstrated for a neutron double-crystal diffractometer using GaAs 200 reflections, resulting in an increase in *Q* resolution by a factor of 6.1 as compared with a standard Si 220 setup.

## 1. Introduction

Pinhole small-angle neutron scattering (SANS) instruments like D11 at the Institute Max von Laue–Paul Langevin (Lindner & Schweins, 2010 ▸) reach a minimum *Q* value of about 3 × 10^−4^ Å^−1^, while ultra-small-angle neutron scattering (USANS) instruments in Bonse–Hart (BH) geometry like S18 (Kroupa *et al.*, 2000 ▸) can access *Q* values one order of magnitude smaller, about 2 × 10^−5^ Å^−1^ (Rauch *et al.*, 1978 ▸; Agamalian *et al.*, 2010 ▸). For many research projects, like the study of hierarchical structures of modern polymers or in food science, for composite construction materials or geological samples relevant for oil extraction and CO_2_ storage or with a self-similar structure extending over many orders of magnitude (Radliński *et al.*, 1999 ▸), or for systems where the size and shape of large objects are of interest such as for biological cells (Semeraro *et al.*, 2022 ▸), it is desirable to have a further extended *Q* range available.

BH instruments are multi-bounce double-crystal diffractometers (DCD) in reflection geometry in a nondispersive setting. A very high structural fidelity of the crystals is a stringent requirement as the reflection profile should be fully determined by dynamic diffraction. Today, Si crystals are used exclusively in BH cameras because of their extremely high structural quality, which has been developed for Si in relation to its fundamental role in the semiconductor industry. Dislocation-free boules with a length well in excess of 1 m and with homogeneous doping became a standard decades ago.

Because the Darwin profile of a Bragg reflection (Darwin, 1914 ▸) has a slow falloff outside the plateau region (approximately 1/*Q*^2^), multiple-bounce crystal slabs are employed to sharpen this decay (Bonse & Hart, 1965 ▸; Schwahn *et al.*, 1985 ▸). While this is significant in the small-*Q* regime to reach rapidly a high contrast between the direct beam and the diffracted beam, the FWHM is only improved slightly.

The *Q* resolution Δ*Q* of a DCD instrument is determined by the Darwin width of the crystal reflection used, while beam divergence both in plane and out of plane can be relaxed to boost intensity. With 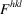 and 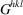 representing, respectively, the crystallographic structure factor and the reciprocal lattice vector of a Bragg reflection *hkl*, the *Q* width for the extension of the full plateau range is given by
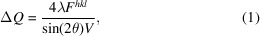
where θ is the Bragg angle and *V* is the volume of the unit cell. We note that the Debye–Waller factor is included in the structure factor 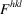. However, it is of minor importance for the present study, and both the Debye–Waller factor and beam absorption are neglected in the following.

Table 1 ▸ shows the theoretical Δ*Q* values of the plateau region (row number 3) and the FWHM of convoluted single-bounce (row 4) and triple-bounce (row 6) profiles for the reflections Si 220 and Si 111. These values are compared in column 5 with those of GaAs 200. The Bragg angles of θ = 30.00° and of θ = 33.69° for Si and GaAs, respectively, correspond to the setting used in the present experiment. Further, Table 1 ▸ includes experimental values (rows 5 and 7) from the present study (in italics).

The salient information from Table 1 ▸ is that the expected *Q* resolution for a DCD with GaAs 200 is superior by about a factor of 10 as compared with Si 220 or Si 111 setups (rows 4 and 6).

The sharper *Q* resolution of GaAs 200 as compared with the Si reflections is related to the small value of the structure factor *F*^200^ which comes about as planes of Ga interfere destructively with planes of As, with both types of planes featuring the same number density of atoms and similar coherent scattering lengths of 6.58 and 7.288 fm for Ga and As, respectively (Sears, 1992 ▸). This low structure factor of GaAs 200 offers the possibility to realize a neutron BH instrument, termed xUSANS in the following, with a significantly improved small-angle performance reaching a real-space resolution of some tenths of a millimetre.

While this length scale is also accessible by other techniques including optical microscopy, it still seems desirable to have neutrons available as an alternative probe to explore specimens by diffraction. Neutron beams exploit specific features like the high sensitivity of neutrons to hydrogen and magnetism, the possibility of contrast variation because of the unique isotope sensitivity, the high penetration power of neutrons to most materials, and the feature of producing a representative average over a large sample volume.

We note that the narrow Darwin width of GaAs 200 as compared with Si 111 has been taken advantage of recently when a significantly improved energy resolution was demonstrated in neutron backscattering spectroscopy (Kuhlmann *et al.*, 2019 ▸).

## 2. Structural fidelity of GaAs

The structural quality of the GaAs crystals is the central issue in the present endeavor. We have used two crystal discs with thickness and diameter of 3 and 50 mm, respectively, cut from the head piece of a commercially grown boule by the company FCM (Freiberger Compund Materials, Germany). Any surface damage from cutting was removed by etching.

**Figure 1:**
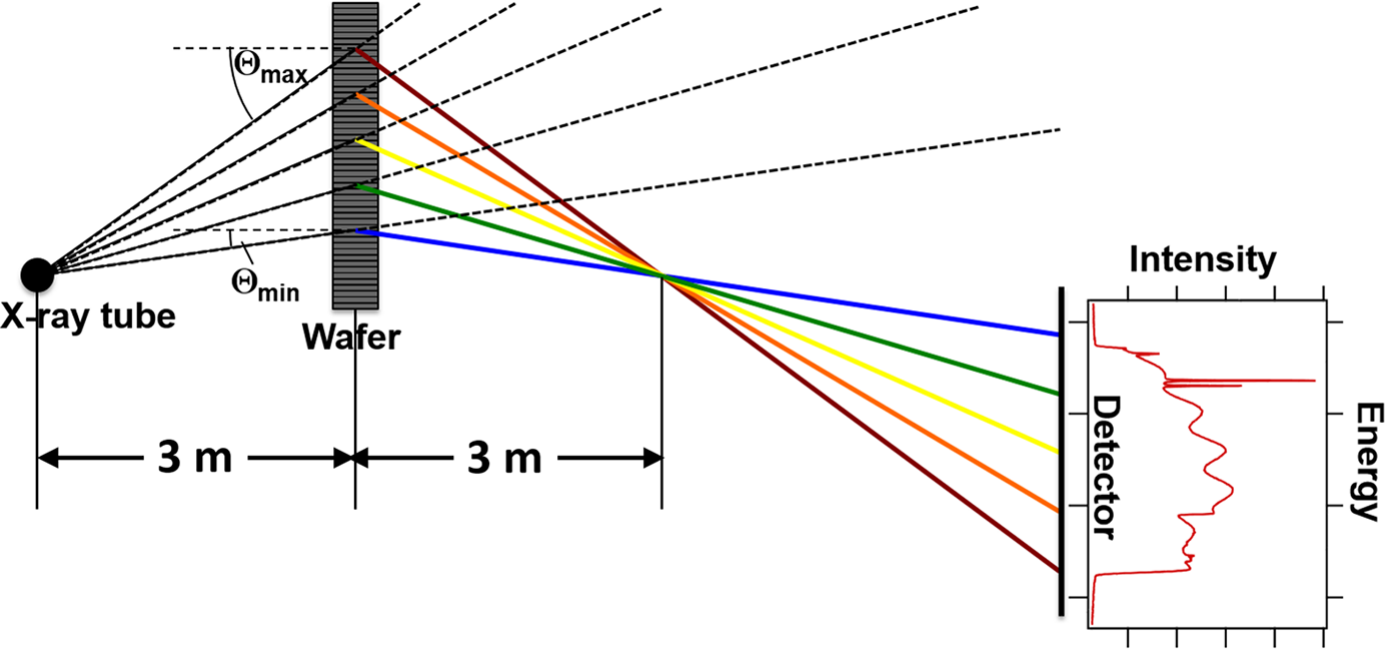
Layout of the in-plane geometry for energy-dispersive X-ray topography in HexBay.

The structural quality was assessed in a first analysis by topographic images taken at the high-energy X-ray laboratory HexBay of the Lehrstuhl für Kristallograpie und Strukturphysik, Universität Erlangen-Nürnberg (Stockmeier & Magerl, 2008 ▸). The divergent polychromatic X-ray beam from a round tungsten anode with a diameter of 0.4 mm and an accelerating voltage of 110 keV hits the entire sample positioned in transmission mode at a distance of 3 m from the X-ray source. This geometry produces a focal point in the diffraction plane (a focal line perpendicular to the diffraction plane) when the source–sample distance equals the sample–detector distance (Fig. 1 ▸).

Moving the area detector further away produces an energy-dispersive topographic image which represents the wavelength-dependent intensity of the source multiplied by the local reflectivity of the crystal.

**Figure 2:**
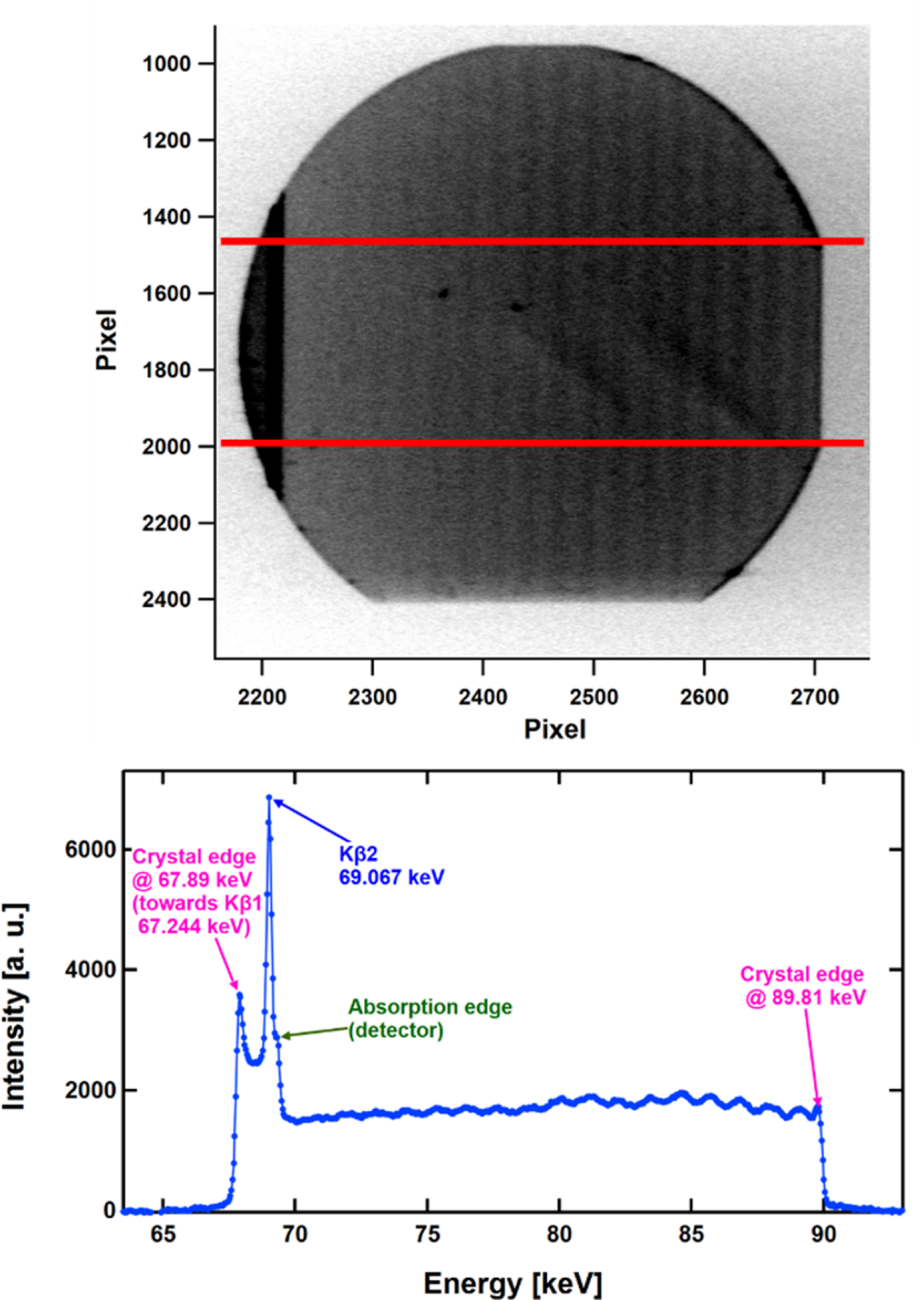
Top: energy-dispersive X-ray topography of a 3 mm-thick GaAs crystal. The MAR area detector is positioned 6 m from the sample (see Fig. 1 ▸). Exposure time 60 min. Bottom: intensity pattern between the two horizontal lines of Fig. 2 ▸ (top) projected downwards to create a line profile. It shows on the left the onset of the characteristic *K*β_1_ line and the characteristic *K*β_2_ line of the tungsten anode followed at higher energies by the intensity pattern of the crystal with *Pendellösung* oscillations.

This is shown in Fig. 2 ▸ (top) for a GaAs crystal doped with Si to a level of 1–2 × 10^18^ cm^−3^, used in the present study. The Si doping suppresses the formation of dislocations during crystal growth. Indeed, only a few dislocations are present, shown by the isolated dark spots in Fig. 2 ▸ (top). Some further crystal defects likely relating to an inhomogeneous convection flow during crystal growth are brought forward by two inclined intensity ridges in the lower right part of the image. However, the salient feature in Fig. 2 ▸ (top) is the presence of *Pendellösung* oscillations, shown by the striped intensity pattern.

These become more evident when projecting the intensity between the two horizontal lines downwards, yielding the line profile in Fig. 2 ▸ (bottom). The high intensity on the left side is attributed to the characteristic *K*β_1_ and *K*β_2_X-ray lines of the tungsten tube, which are useful for the energy calibration as shown. The energy-dependent *Pendellösung* oscillations are proof that the scattering properties of this crystal are governed by dynamic diffraction with coherent wave states building up in the entire crystal volume.

## 3. GaAs 200 small-angle setup

The standard USANS S18 setup features a triple-bounce Si 220 in the primary beam at a Bragg angle of 30°. A second crystal of identical quality is located on a high-resolution rotation and tilt stage (Hainbuchner *et al.*, 2000 ▸). For reasons of radioprotection, it is not possible at present to replace the first Si in the primary protection of S18 by GaAs. In consequence, we used an ideal Si 111 slab as a pre-monochromator in the primary beam with a *d* spacing of 3.1356 Å, which is close to the *d* spacing of GaAs 200 = 2.8267 Å. The wavelength λ = 3.1356 Å delivered by this pre-monochromator implies a Bragg angle of 33.687° at the GaAs crystals. The mismatch in *d* spacing causes a loss in intensity by a factor of about 70. Inherent further losses by about one order of magnitude result from the narrow Darwin width of GaAs 200 as compared with Si 220 and by a factor of 2 from the flux in the thermal primary beam of S18 which is halved between 1.9 Å (Si 220 setup) and 3.1 Å (GaAs 200 setup). In consequence, the expected count rate in this feasibility study is reduced by a factor of about 1400, which compares reasonably well to a measured reduction of 1230. We note that the count rate would only reduce by a factor of 10 in a dedicated instrument.

**Figure 3:**
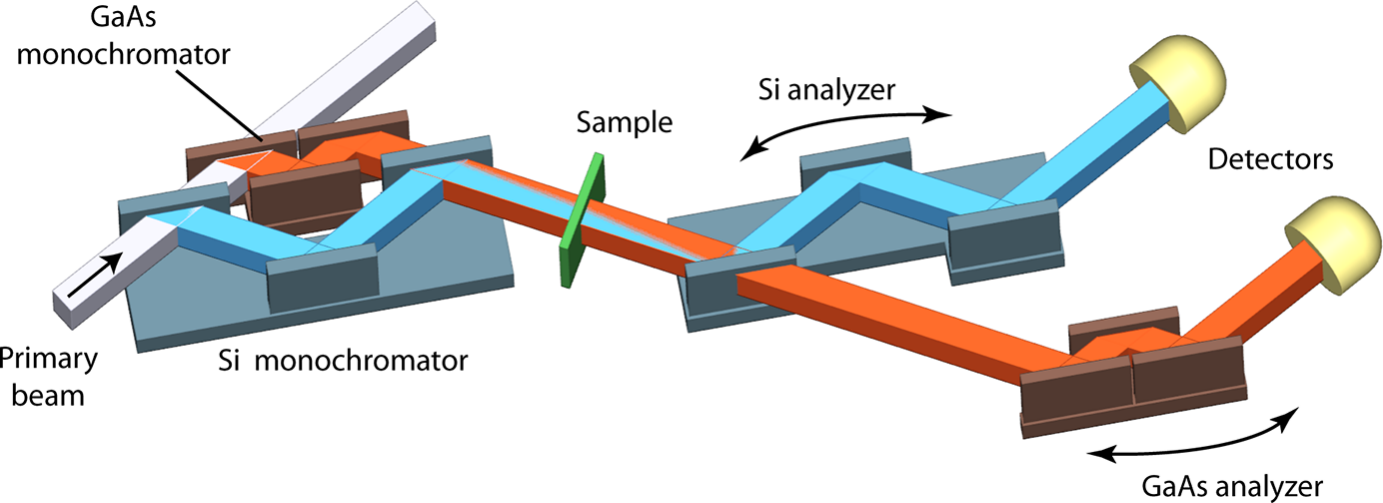
Layout of a BH diffractometer with a nested Si (*e.g.* Si 220) and a GaAs 200 monochromator providing an identical beam path to the sample and with two separate analyzer crystals and detectors.

As a consequence of the narrow Darwin width, the reflected intensity of the GaAs crystal may render high-*Q* measurements difficult. In this context, the GaAs setup should be considered as an extension for standard Si setups and not as a replacement. It is possible to make a nested setup which allows one to measure the low-*Q* extension by GaAs and, simultaneously, the standard *Q* range by Si crystals. A corresponding layout is sketched in Fig. 3 ▸. The primary beam falls onto a nested pair of Si 220 (or similar) and GaAs 200 crystals. For a Bragg angle of 30°, the crystals create on the identical beam path two wavelengths of 1.9 Å (shown in blue) and 2.8 Å (shown in orange) from Si 220 and GaAs 200, respectively. Downstream of the sample, two corresponding analyzer crystals are placed on individual rotation stages to record simultaneously USANS data with the Si crystals and xUSANS data with GaAs 200.

Scaling and merging of scattered intensities from the two *Q* ranges of a GaAs 200 xUSANS and a traditional Si-based USANS diffractometer measured with a slightly overlapping *Q* region should be straightforward, requiring only one scaling factor. USANS data in general will be measured over a *Q* range of a few orders of magnitude and up to high *Q* values where intensities may become weak. In contrast, the simultaneous measurement with xUSANS will only extend the low-*Q* range by one order of magnitude where scattered intensities are high. Both these effects should compensate the reduction of the primary flux in xUSANS by a factor of 10. In consequence, it is expected that no extension of beamtime will be needed.

A further benefit of a nested monochromator stems from the fact that the beam paths up to the sample are coincident (Fig. 3 ▸) and the identical sample is being measured, requiring no further corrections.

For the present setup the first GaAs crystal was placed on the usual stage of the second Si 220 crystal of S18 and a similar setup was installed for the second GaAs crystal. The rotation stages were piezo driven and controlled by absolute encoders with 10^−5^ ° resolution. The tilt was controlled in steps of 10^−3^ °. Finally, detectors for the transmitted and diffracted beams were appropriately positioned.

The GaAs crystals were placed with their flats on a soft rubber band and gently clamped at the top to ensure mechanical stability during scan movements while avoiding at the same time any mechanical strain.

## 4. Results and discussion

**Figure 4:**
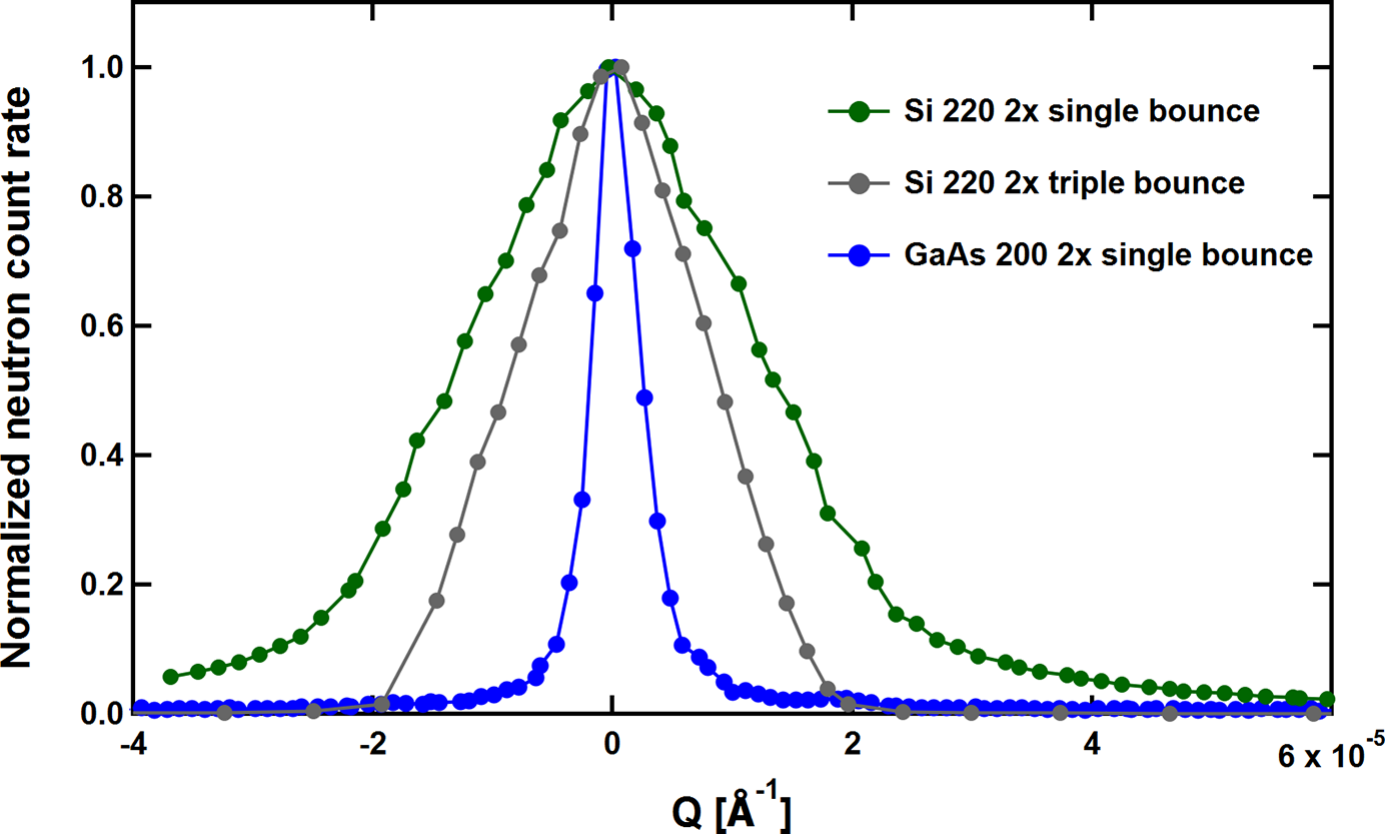
Resolutions of DCD setups measured with single-bounce perfect Si 220 crystal slabs (green) and single-bounce GaAs 200 discs (blue). In addition, the resolution with a triple-bounce Si 220 (standard USANS setup of S18) is shown in gray.

Fig. 4 ▸ shows the measured resolution functions, *i.e.* the rocking pattern of the intensity of the empty DCD. Green and blue dots represent the data for the single-bounce setups of Si 220 and GaAs 200, respectively. The FWHM of GaAs 200, 0.46 × 10^−5^ Å^−1^, corresponding to a real-space dimension of 136 µm, is 6.1 times sharper than the value for Si 220, 2.79 × 10^−5^ Å^−1^ (row 5 in Table 1 ▸), corresponding to a real-space dimension of 23 µm. This demonstrates the potential of GaAs 200 in realizing an xUSANS instrument with a significantly extended small-*Q* range. While the demonstrated Si 220 resolution meets the expectation for an ideal BH camera, we note that the measured GaAs resolution is about a factor of 1.6 lower with respect to the theoretical limit of ideal crystal diffraction. The origin of this broadening is the presence of some crystal defects as indicated in the X-ray topography in Fig. 2 ▸.

For completeness, Fig. 4 ▸ also displays as gray dots the USANS resolution of S18 in its standard triple-bounce Si 220 mode. The FWHM of 1.86 × 10^−5^ Å^−1^ (row 7 in Table 1 ▸), corresponding to a real-space dimension of 34 µm, is smaller by 35% compared with the single-bounce geometry, and the line shape approaches a favorable triangular profile with a significant reduction of the trailing wings (Bonse & Hart, 1965 ▸). Obviously, multiple-bounce reflections are to be envisaged when intending to make available an application-oriented diffractometer.

**Figure 5:**
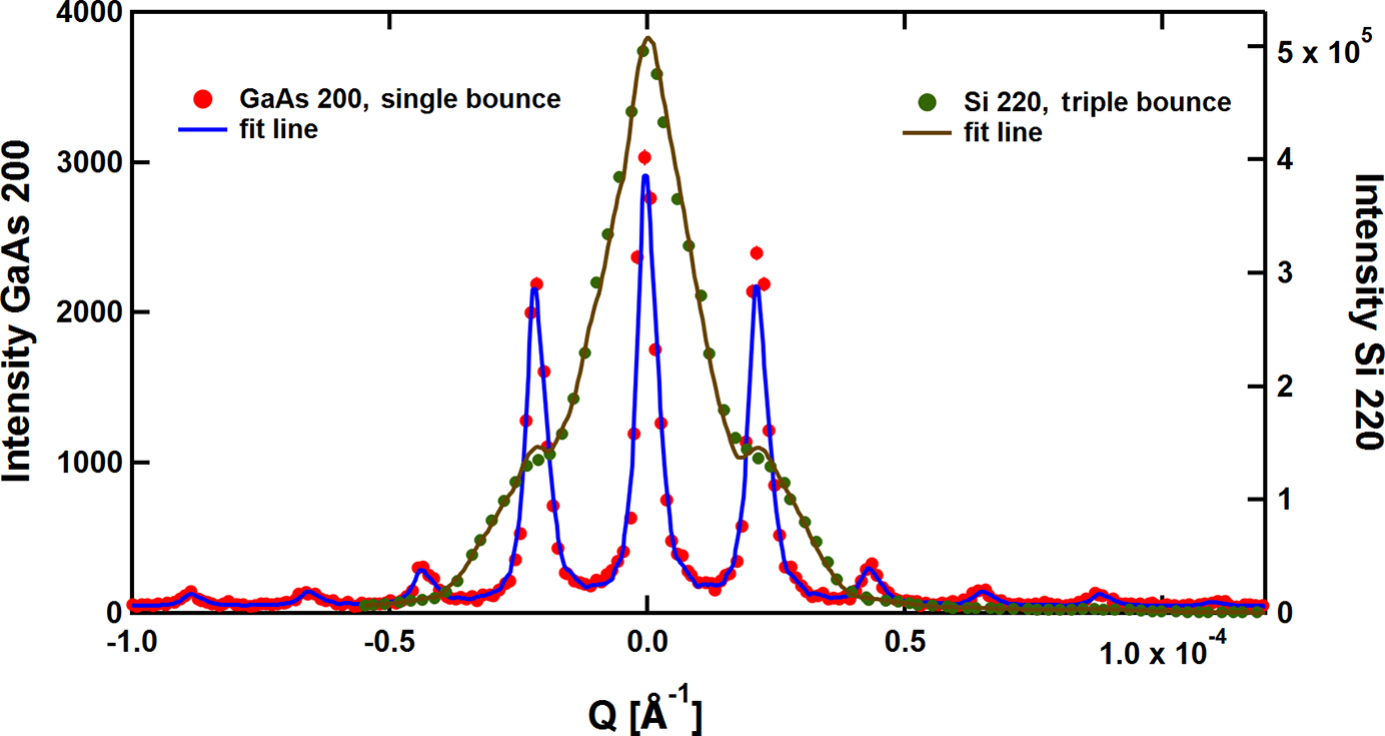
Small-angle pattern of an etched grid with a nominal periodicity of 28 µm (Trinker, 2006 ▸) measured with two triple-bounce Si 220 crystals (green dots and brown fitting line) and with two single-bounce GaAs 200 crystal slabs (red dots with blue fitting line).

To demonstrate the potential of GaAs 200 reflections for very small *Q* measurements, we compare in Fig. 5 ▸ data of an etched grating on a Si wafer with grooves 40 µm deep, 8 µm wide, a nominal periodicity of 28 µm and total specimen size 2 × 2 cm (Trinker, 2006 ▸). This sample area was identical in all cases and it is typical for a USANS instrument like S18 located at a neutron guide.

**Figure 6:**
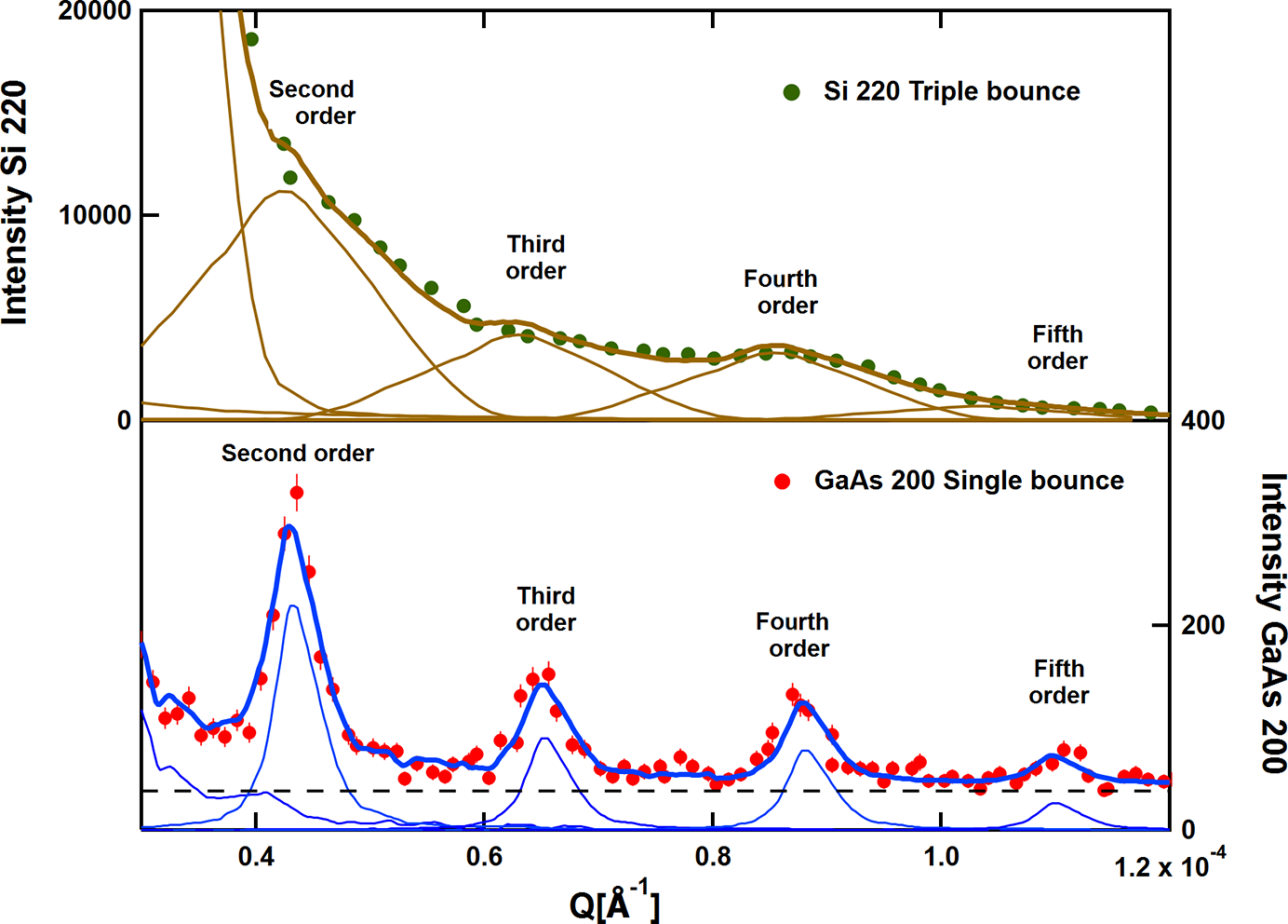
The xUSANS pattern between 0.3 × 10^−4^ and 1.2 × 10^−4^ Å^−1^, *i.e.* in the range from the second to the fifth order of diffraction, taken with Si 220 (top) and GaAs 200 (bottom). See text for details.

The data were taken in the standard S18 mode with triple-bounce Si 220 (green dots) and with the present feasibility setup with two single-bounce GaAs 200 slabs (red dots). The fits of the data have been performed using the experimentally measured resolution. They are shown in Fig. 5 ▸ and Fig. 6 ▸ with brown lines for Si 220 and blue lines for GaAs 200. Clearly, shoulders are visible in the Si 220 setup relating to the grating. However, the GaAs 200 excels, its superior performance revealing well separated diffraction peaks.

A magnified plot of the xUSANS pattern is shown in Fig. 6 ▸ from 0.3 × 10^−4^ to 1.2 × 10^−4^ Å^−1^. In both cases, a description of the data requires diffraction peaks up to the fifth order. The contributions to the individual orders are shown by thin lines according to the color scheme as in Fig. 5 ▸, and the sums are shown by thick lines. In addition, a constant background has been considered, shown by the dashed black line in the case of GaAs 200. This component is not visible in the case of Si 220 due to the higher count rate, as mentioned earlier.

**Figure 7:**
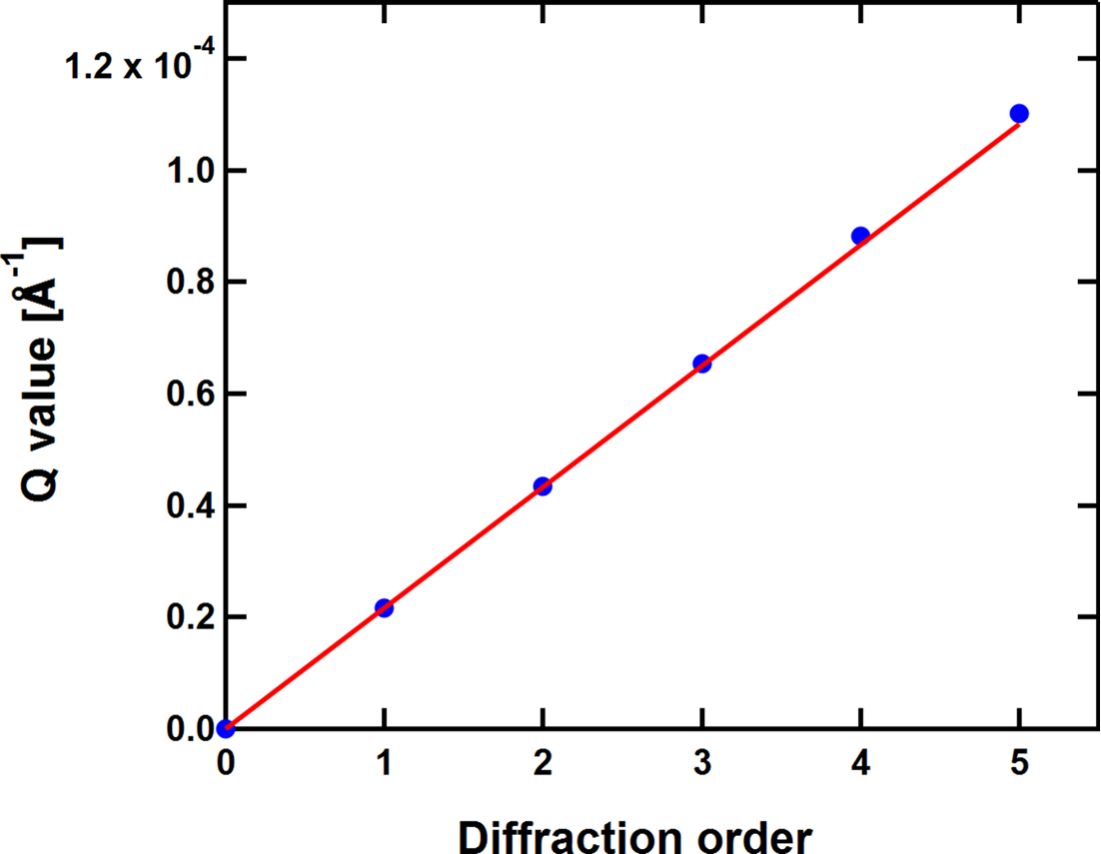
*Q* values and order of diffraction for GaAs 200. Error bars are smaller than the symbols.

The breakdown into the various diffraction orders is vague in the case of Si 220, whereas it is well defined in the case of GaAs 200. The positions of the individual diffraction orders have been considered as free parameters for the fitting in order to have a check on the quality of the setup (although they are strictly related to each other). The result for GaAs 200 is shown in Fig. 7 ▸. A linear fit yields a periodicity of 29.01 ± 0.02 µm, in agreement with an earlier assessment by electron microscopy which gave a periodicity of 28 µm (de Haan *et al.*, 2007 ▸, Trinker, 2006 ▸).

## 5. Summary

For reasons relating to its small crystallographic structure factor, the GaAs 200 reflection is a promising candidate to realize a BH DCD offering for triple-bounce crystals access to a *Q* regime down to an FWHM = 2 × 10^−6^ Å^−1^. This corresponds to real-space structures of 300 µm, which have so far been inaccessible to neutron diffraction. Although we are well aware that such length scales are readily accessible by other techniques, notably optical microscopy, it still may be desirable to have neutron diffraction with its different scattering cross sections and unique properties like contrast matching or isotope sensitivity and sample transparency at hand as well.

In the present study by high-energy X-ray topography Si-doped crystals of GaAs showed well developed *Pendellösung* oscillations, proving that diffraction is extinction dominated, a mandatory requirement to realize a high-quality BH instrument. A comparison of FWHM resolutions in DCD setups between single-bounce Si 220 and the here-investigated GaAs 200 shows an improvement in *Q* resolution by a factor of 6.1. A further gain by a factor of 2.1 appears possible when the ideal crystal limit in a triple-bounce setup can be realized by further scrutinizing the possibilities in crystal growth and handling, promising a total extension of the *Q* range by one order of magnitude compared with the present-day performance of neutron BH diffractometers.

We note that GaAs is not the only choice for improved xUSAS instruments both for neutrons and X-rays. Any reflection with a low structure factor can be used, provided that crystals of sufficient size and of high structural quality are available.

## Figures and Tables

**Table 1:** Crystallographic parameters and *Q* widths of individual reflections and for convoluted multi-bounce settings compared with experimental values from this study

	Reflections and settings	Si 220 (θ = 30°)	Si 111 (θ = 30°)	GaAs 200 (θ = 33.69°)
1	Structure factor |*F*^*hkl*^| (fm)	33.19	23.47	2.83
2	*G*^*hkl*^ (Å^−1^)	3.272	2.003	2.222
3	Δ*Q* (plateau region) (Å^−1^)	1.83 × 10^−5^	2.12 × 10^−5^	0.20 × 10^−5^
4	Δ*Q* (FWHM) convoluted single-bounce (Å^−1^)	2.57 × 10^−5^	2.97 × 10^−5^	0.29 × 10^−5^
*5*	*Experimental*	*2.79 × 10^−5^*		*0.46 × 10^−5^*
6	Δ*Q* (FWHM) convoluted triple-bounce (Å^−1^)	1.95 × 10^−5^	2.25 × 10^−5^	0.22 × 10^−5^
*7*	*Experimental*	*1.86 × 10^−5^*		

## Data Availability

Data are available under MAGERL Andreas; APPEL Markus; LEMMEL Hartmut; and ZOBEL Mirijam (2023). Extended *Q* range in USANS with GaAs 200. Institut Laue–Langevin (ILL) doi:10.5291/ILL-DATA.CRG-3068, https://doi.ill.fr/10.5291/ILL-DATA.CRG-3068
